# Comparison of Urine Dipstick and Urine Sediment Microscopy With Culture for Diagnosing Pediatric Urinary Tract Infections: A Retrospective Cross-Sectional Study at Tikur Anbessa Hospital, Ethiopia

**DOI:** 10.1155/ijpe/6690751

**Published:** 2025-10-23

**Authors:** Tajudin Adem, Damte Shimelis, Tesfaye Negasa, Melkamu Siferih

**Affiliations:** ^1^Department of Pediatrics and Child Health, College of Medicine and Health Sciences, Addis Ababa University, Addis Ababa, Ethiopia; ^2^Division of Pediatric Nephrology, Department of Pediatrics and Child Health, College of Medicine and Health Sciences, Addis Ababa University, Addis Ababa, Ethiopia; ^3^Department of General Surgery, Medawolabu General Hospital, Dodola, Oromia Region, Ethiopia; ^4^Department of Obstetrics and Gynecology, School of Medicine, Debre Markos University, Debre Markos, Amhara Region, Ethiopia

**Keywords:** Ethiopia, pediatric urinary tract infections, urine culture, urine dipstick, urine sediment microscopy

## Abstract

**Background:**

Urinary tract infections are common in children, and early diagnosis is crucial to prevent long-term complications such as renal scarring. While urine culture remains the gold standard, its 24–72-h turnaround time can delay treatment. To expedite diagnosis, rapid tests such as urine dipstick and microscopy are commonly used, but their reliability in resource-constrained settings like Ethiopia is uncertain. This study is aimed at comparing the diagnostic performance of these rapid tests with urine culture in pediatric patients.

**Methods:**

A retrospective cross-sectional study was conducted at Tikur Anbessa Specialized Hospital, Ethiopia, from September 2021 to September 2022. The diagnostic performance of urine dipstick tests (nitrites and leukocyte esterase) and urine microscopy for white blood cells (WBCs) was compared to urine culture results, with sensitivity, specificity, PPV, NPV, and diagnostic accuracy calculated.

**Results:**

A total of 224 children (mean age 5 years; 50% < 4 years) were included in the final analysis. Dipstick nitrite positivity was low (10.9%), limiting its reliability. Leukocyte esterase positivity was higher in males (56%) than in females (43.2%), with an overall rate of 51.8%. Urine WBC microscopy showed higher positivity in males (52.7%) than in females (35.1%). Urine culture had a higher positivity rate in females (55.4%) than in males (47.3%), with an overall rate of 50%. Dipstick nitrite had high specificity (94.6%) but low sensitivity (16.1%), while leukocyte esterase had moderate sensitivity (59.8%) and specificity (75.9%). Combining dipstick tests improved sensitivity (61.6%) but reduced specificity. Urine WBC microscopy showed 67.0% sensitivity, 73.2% specificity, and 70.1% accuracy, with a 33% false-negative rate.

**Conclusion:**

Dipstick nitrites and leukocyte esterase offer a rapid, cost-effective way to detect UTIs in children and guide early treatment, but their limited specificity necessitates confirmatory tests like urine culture or WBC microscopy. Future research should explore novel urinary biomarkers to improve accuracy, minimize invasiveness, and account for age- and sex-specific differences.

## 1. Introduction

Urinary tract infections (UTIs) are among the most prevalent bacterial infections in pediatric populations, with substantial effects on both immediate health outcomes and long-term renal function [1, 2]. A pediatric UTI is typically characterized by the presence of a pathogen in the urine, confirmed either through a positive culture or by clinical symptoms accompanied by supporting laboratory findings [3]. In children, UTIs are a major cause of morbidity, particularly in younger age groups, and, if left untreated or inadequately managed, can lead to complications such as renal scarring, hypertension, and chronic kidney disease. Early detection and appropriate management are crucial for preventing such adverse outcomes [4–8].

The diagnosis of UTIs in children typically relies on a combination of clinical evaluation and laboratory tests [9]. Traditionally, urine culture has been considered the gold standard for UTI diagnosis due to its high sensitivity and specificity [10, 11]. However, urine culture is time-consuming and labor-intensive, often requiring several days to yield results [12]. This has led to a growing interest in alternative diagnostic methods that can provide rapid results, thereby facilitating timely interventions [13, 14].

Urine dipstick tests and urine sediment microscopy are commonly used as initial screening tools for UTIs. The urine dipstick is a rapid, noninvasive test that detects the presence of leukocyte esterase (LE) and nitrites, both of which are indicative of bacterial infection [13, 15, 16]. Although widely used in clinical settings, the accuracy of the dipstick test can be influenced by several factors, including the type of bacteria and the timing of the urine sample collection [17]. On the other hand, urine sediment microscopy involves the microscopic examination of urine for the presence of white blood cells (WBCs), bacteria, and other abnormal elements. This method is cost-effective and provides valuable information about the inflammatory response in the urinary tract. However, its sensitivity and specificity in pediatric populations are still debated [15, 18]. Moreover, recent evidence suggests that alternative staining techniques, such as the revived Sternheimer stain, may further enhance the diagnostic utility of microscopy in detecting UTIs [19].

Despite their widespread use, the diagnostic performance of urine dipstick and microscopy in comparison to urine culture remains a subject of ongoing investigation [20–23]. Several studies have reported varying levels of diagnostic accuracy for these methods, with some suggesting that they may be reliable alternatives to urine culture in specific clinical settings [10, 22, 24–27]. However, the diagnostic accuracy of these tests in pediatric patients, especially in resource-limited settings such as Ethiopia, remains insufficiently studied [2, 28].

This study is aimed at evaluating the diagnostic performance of urine dipstick and urine sediment microscopy for diagnosing UTIs in children by comparing their sensitivity, specificity, and predictive values to urine culture.

The findings are expected to enhance the growing body of literature on the diagnostic accuracy of these rapid tests, particularly in resource-limited African settings, where healthcare resources are scarce and the burden of infectious diseases is high [29, 30]. Ultimately, this research could inform improved diagnostic strategies, providing reliable alternatives to culture, reducing healthcare costs, and minimizing the risk of complications from delayed diagnosis and treatment, thereby improving the quality of care and patient outcomes for children with UTIs.

## 2. Methods and Materials

### 2.1. Study Area, Period, and Design

A hospital-based retrospective cross-sectional study was conducted in the Pediatrics and Child Health Department at Tikur Anbessa Specialized Hospital (TASH) from September 2021 to September 2022. Established in 1964, TASH is the largest and most advanced referral hospital in Ethiopia, affiliated with Addis Ababa University's School of Medicine. It serves as a critical teaching hospital, providing specialized clinical services unavailable at other public or private institutions in the country. With over 800 beds, TASH offers comprehensive diagnostic and therapeutic services, catering to 370,000–400,000 patients annually. The Pediatrics Department, a cornerstone of the hospital, includes more than 240 beds, 9 inpatient units, 11 subspecialty clinics, and a central triage. Patients are initially assessed at triage sites, including the casualty ward, neonatal ICU, and central triage, before being directed to appropriate subspecialty clinics or admitted for further management. The department is staffed by a dedicated team of pediatric residents, subspecialty fellows, and experienced subspecialists, providing expert care across a wide range of pediatric conditions.

### 2.2. Population

The source population comprised all pediatric patients admitted to the Pediatrics Department at TASH during the study period. The study included children aged ≤ 15 years admitted to the casualty or pediatric wards with clinically suspected UTIs or sepsis. For all suspected cases, urine samples were collected at admission before the start of empirical antibiotics, and all four diagnostic tests—dipstick nitrites, dipstick leukocytes, WBC microscopy, and urine culture—were systematically performed, enabling a thorough evaluation of diagnostic performance.

### 2.3. Sample Size Determination and Sampling Technique

To determine the sample size, a 95% confidence level and a 5% margin of error were used, corresponding to a *Z* score of 1.96 from the standard normal distribution curve. The estimated prevalence of UTIs in pediatric patients was 15.8%, based on a previous study at Yekatit 12 Hospital Medical College in Addis Ababa [28]. The initial sample size was calculated to be 204. To account for adjustment for nonresponse or incomplete data, the final adjusted sample size was increased to 224, ensuring sufficient statistical power and minimizing the impact of nonresponses or incomplete data, thus strengthening the reliability and validity of the study. The sampling technique employed was consecutive sampling, where medical records of pediatric patients admitted to the emergency and inpatient pediatric wards, who met the inclusion criteria, were reviewed sequentially for inclusion. Patient charts and laboratory logbooks were retrieved from the medical and laboratory archives consecutively until the target sample size was achieved. This approach ensured a comprehensive and unbiased selection of participants meeting the study's criteria.

### 2.4. Study Variables

Age, sex, and urine culture served as dependent variables, whereas urine dipstick nitrite, LE, and microscopic urine WBC count were considered independent variables.

### 2.5. Data Collection Tools, Data Collection Techniques, and Data Quality Assurance

A structured English version data abstraction checklist was developed by using different published articles [16, 26, 27, 31, 32]. Data was compiled by examining laboratory microbiology logbooks alongside medical records, including both charts and iCare systems. Two BSC nurses with expertise in pediatric care and one medical doctor were recruited for data collection and supervision, respectively. Data collectors were given training by the principal investigator about the goal of the research and data collection procedures. The checklist was pretested on 5% of patients 1 week before the main data collection. Patients in the same area were included in the pretest. The checklist was modified for clarity, sensitivity, and completeness based on the pretest data. The quality of data was assessed every day after data collection.

### 2.6. Specimen Collection, Transportation, Identification, and Quality Control

Urine samples were tested for nitrites and LE using the *Laboquick* Multiple Reagent Strip (E & E Medical Lab, Brighton, United Kingdom) per manufacturer's instructions. LE was read after 2 min, with trace or higher recorded as positive. Nitrite results were interpreted at 2 min and classified as positive or negative based on color change [33, 34]. Urine specimens were inoculated onto MacConkey and blood agar using a sterile 0.002 mL loop and incubated aerobically at 37°C for 24 h. Culture was considered positive at ≥ 10^5^ cfu/mL, negative at < 10^5^ cfu/mL, and contaminated if mixed growth was observed. Samples were centrifuged at 1800 rpm, and WBCs were counted microscopically at 40×; ≥ 5 WBCs/hpf in males or ≥ 10 WBCs/hpf in females were classified as positive [33, 35, 36]. The results were interpreted by qualified laboratory technologists and microbiologists at the time of testing, according to the manufacturer's standardized guidelines and the laboratory's routine protocols. The laboratory maintains standard operating procedures (SOPs) to ensure consistent interpretation of results, and all personnel performing these tests were trained according to these SOPs.

### 2.7. Data Management and Analysis

After data entry and cleaning, statistical analysis was conducted using SPSS Version 26. Data were summarized, tabulated, and analyzed using descriptive statistics, including frequencies and percentages. The correlation between individual dipstick test components and their combinations was assessed in comparison to urine culture results. The diagnostic performance of dipstick test components and urine culture was evaluated using sensitivity, specificity, positive predictive value (PPV), and negative predictive value (NPV), with urine culture serving as the gold standard. Cross-tabulation was employed to compare urine dipstick test results with urine WBC counts observed under the microscope, while Pearson correlation was used to determine the strength of their relationship. The final results were presented in simple statistical tables.

## 3. Results

During the study period, 2166 pediatric patients were admitted, of whom 1300 submitted urine samples for different urine analyses. A total of 224 children (1 month–15 years; mean age 5 years) who completed all four diagnostic tests (dipstick nitrite, dipstick LE, microscopic WBC examination, and urine culture) were included in the final analysis. The cohort was predominantly male, comprising 150 (66.7%) males and 74 (33.3%) females, with 50% of the children under the age of 4. Urine samples were obtained at admission, before the initiation of empirical antibiotics. Dipstick nitrite positivity, a common screening test for UTIs, was low across both genders, with an overall prevalence of 10.9% for both males and females, suggesting that nitrite detection may not be a reliable marker in this age group. In contrast, dipstick LE positivity, indicative of the presence of WBCs in the urine, was significantly higher in males (56%) compared to females (43.2%), yielding an overall positivity rate of 51.8%.

Further examination of urine WBC microscopy demonstrated that males had a greater positivity rate (52.7%) than females (35.1%), contributing to an overall positivity rate of 47.1%. These results align with the dipstick LE findings, reinforcing the notion that males in this cohort may be more likely to exhibit urinary leukocyturia, a key indicator of UTI or urinary inflammation. Interestingly, urine culture results, which serve as the gold standard for diagnosing UTIs, demonstrated a higher positivity rate in females (55.4%) compared to males (47.3%), resulting in an overall culture positivity rate of 50% (Table 1).

Using urine culture as the gold standard, this study evaluated the diagnostic performance of dipstick LE and nitrite—both individually and in combination—for detecting UTIs. The findings highlight a critical trade-off between sensitivity and specificity.

Nitrite alone demonstrated high specificity (94.6%; 95% CI: 89.2%–97.4%) but extremely low sensitivity (16.1%; 95% CI: 14.1%–23.8%), indicating that while a positive result strongly suggests infection (PPV = 75%), a negative result fails to reliably exclude it (NPV = 53%). In contrast, LE exhibited moderate sensitivity (59.8%; 95% CI: 57.2%–66.7%) and specificity (75.9%; 95% CI: 67.8%–82.3%), providing a more balanced but still imperfect diagnostic tool (accuracy = 67.9%; 95% CI: 60.3%–72.1%). When both markers were positive, specificity remained high (94.6%; 95% CI: 88.7%–98.2%); however, sensitivity dropped significantly to 14.3% (95% CI: 12.4%–17.1%), increasing the risk of missed infections (accuracy = 54.5%; 95% CI: 48.5%–58.4%). Notably, when either nitrite or LE was positive, sensitivity improved to 61.6% (95% CI: 58.6%–67.9%), offering a more inclusive but less specific diagnostic approach (Table 2).

The findings presented in Table 3 indicate that urine WBC microscopy demonstrated a sensitivity of 67.0% (95% CI: 62.9%–73.6%) and a specificity of 73.2% (95% CI: 68.1%–79.5%), correctly identifying 67% of culture-confirmed UTI cases while failing to detect the infection in 33% of cases. The PPV was 71.4%, meaning that among WBC-positive cases, there was a 71.4% probability of a true UTI. Conversely, the NPV was 68.9%, indicating that 68.9% of WBC-negative cases were truly infection-free. The overall diagnostic accuracy was 70.1% (95% CI: 65.0%–77.4%), reflecting moderate reliability in detecting UTIs. However, the 33% false-negative rate highlights the risk of missed infections, potentially leading to delayed treatment and associated complications. Additionally, the 27.9% false-positive rate suggests that reliance on WBC detection alone may contribute to unnecessary antibiotic use, increasing the risk of antimicrobial resistance.

The next table (Table 4) provides a comparative evaluation of the LE dipstick test against microscopy for detecting WBCs. This assessment highlights the diagnostic accuracy of the LE dipstick, examining its sensitivity and specificity. By comparing dipstick results with microscopy—the reference standard—the table offers valuable insights into the test's reliability, its strengths in ruling out WBC presence, and its limitations in detecting all true-positive cases. The LE dipstick test demonstrates high specificity (92.5%), effectively ruling out WBC presence in most true-negative cases. However, its moderate sensitivity (80.8%) raises concerns, as it failed to detect WBCs in 19.2% of microscopy-confirmed positive cases, leading to a significant false-negative rate. This suggests a potential risk of underdiagnosing infections or inflammatory conditions when the dipstick is used as a standalone test. On the other hand, its low false-positive rate (7.5%) enhances its reliability in confirming the absence of WBCs, minimizing the likelihood of unnecessary treatments.

Table 5 presents the Pearson correlation between LE dipstick results and WBC count per high-power field (hpf) in microscopic examination. The correlation coefficient (*r* = 0.741) indicates a strong positive correlation between the two methods, suggesting that as dipstick leukocyte levels increase, WBC counts in microscopy also tend to rise. This correlation is statistically significant (*p* < 0.01), meaning the relationship is unlikely to have occurred by chance.

## 4. Discussion

This study comprehensively evaluated the diagnostic accuracy of urine dipstick parameters—LE and nitrite—alongside urine microscopy, using urine culture as the definitive gold standard for pediatric UTI diagnosis. Our findings highlight notable limitations in dipstick-based screening, particularly the poor sensitivity of nitrite (16.1%) despite its high specificity (94.6%). This low sensitivity is especially problematic in pediatric populations. In contrast, LE demonstrated moderate sensitivity (59.8%) and specificity (75.9%), reflecting a performance similar to that of urine microscopy for WBCs, which showed sensitivity of 67.0% and specificity of 73.2%, and moderate accuracy of 70.1%. Additionally, while combining nitrite and LE improved sensitivity slightly (61.6%), it came at the cost of specificity. In our cohort, 50% of children were culture positive; however, this figure should not be interpreted as the true prevalence of pediatric UTI in the admitted population. Because only children who underwent all four diagnostic tests were included and many were clinically suspected of UTI, the proportion likely reflects selection bias. Rather, this high culture positivity rate provides context for evaluating the diagnostic performance of dipstick and microscopy in this study.

A notable sex-based discrepancy was observed, with males exhibiting higher LE (56%) and WBC (52.7%) positivity, while culture-confirmed UTIs were more prevalent in females (55.4%). This discrepancy between male and female UTI patterns becomes clearer when we consider the factors underlying these differences. Our findings indicate that males had higher LE (56%) and WBC (52.7%) positivity, despite a lower prevalence of culture-confirmed UTIs. This could be attributed to factors such as anatomical differences, immune response modulation, or circumcision status, as noted in previous studies [31, 32, 37–40]. In contrast, females exhibited a higher prevalence of culture-confirmed UTIs, which is consistent with established research linking a shorter urethra to an increased risk of ascending infections [41, 42]. While females are more susceptible to UTIs due to these anatomical factors, males might experience more chronic infections with greater morbidity, potentially influenced by differences in immune responses and pathogen virulence [32]. These findings underscore the need for sex-specific diagnostic and treatment strategies, considering both anatomical and immunological differences that could significantly affect disease outcomes.

Our findings align with prior research emphasizing the limited diagnostic reliability of urine dipstick tests in pediatric UTIs, largely due to the poor sensitivity of nitrite. Studies have consistently reported low nitrite sensitivity (ranging from 23% to 49%), reinforcing its restricted applicability in young children [9, 16, 27, 43–46]. This limitation is primarily attributed to the rapid urine turnover in young children, which prevents sufficient nitrite accumulation by uropathogens such as *Escherichia coli* [24, 27]. Consistent with this trend, our study found a nitrite sensitivity of 16.1% and specificity of 94.6%, closely matching the findings of Coulthard et al. [47], who reported a sensitivity of 23% and specificity exceeding 90%. These results further underscore the test's limited utility as an independent diagnostic tool. However, our reported nitrite sensitivity is notably lower than that of other studies, which have documented sensitivities of 69.7% [25], 77.1% [48], 85% [45], and 99.5% [27]. The observed variation may be primarily attributed to the fact that 50% of the children in our study are under the age of 4. Additionally, other contributing factors may include a combination of age-related physiological differences, variations in uropathogen profiles, limited dietary nitrate intake, differences in urine sample collection timing, alterations in urinary pH, disparities in test methodologies, and the potential influence of prior antibiotic exposure. The overall implication is that a significant number of UTIs may be overlooked, particularly in children with atypical presentations or infections caused by non*–E. coli* pathogens.

Additionally, the high specificity of the nitrite dipstick test (94.6%) suggests that a negative result is a reliable means of excluding UTIs in children, thereby potentially minimizing the need for further diagnostic procedures or unnecessary treatment. This positions the nitrite test as a valuable diagnostic tool in clinical practice, particularly given its superior specificity compared to both the LE dipstick test and urine microscopy.

Similarly, LE demonstrated moderate diagnostic performance in our study, with a sensitivity of 59.8% and a specificity of 75.9%, comparable to previous findings that range between 48% and 70% sensitivity and 70% and 88% specificity [49–51]. Our findings show lower sensitivity and specificity compared to previous studies, which reported values such as 92% sensitivity and 96.4% specificity [27], 77.1% sensitivity and 94.5% specificity [48], and 95% sensitivity with 98% specificity [52]. Several factors likely account for the variations in sensitivity and specificity observed in our study. The age of the children may influence test outcomes, as younger children may have distinct immune responses and susceptibility to UTIs, which can affect the accuracy of the test. Additionally, the method of urine collection is a critical factor—studies employing more standardized techniques like clean-catch, first-voided, or suprapubic samples typically yield more reliable results. In contrast, the random urine samples collected in our study could result in greater variability in LE concentrations, affecting diagnostic performance. These findings underscore the need to adapt diagnostic strategies based on both age and urine collection methods to improve clinical precision in pediatric care.

Our results align with previous research indicating that combining nitrite and LE enhances sensitivity but reduces specificity, highlighting a fundamental trade-off between diagnostic efficiency and reliability [10, 21, 26, 27, 50, 53]. The modest improvement in sensitivity (61.6%) observed with the combination of LE and nitrite in our study aligns with previous research, which reports a sensitivity range of 60%–70% for dual-marker testing, although this comes with an increased risk of false positives. However, when either of the dipstick markers was evaluated, both the sensitivity and accuracy were comparable to urine WBC microscopy. These findings suggest that the combination of both dipstick markers can be a valuable tool for ruling in UTIs. However, it also highlights their limitations in effectively ruling out UTIs, particularly in symptomatic children. Consequently, this approach may be more suitable for initial screening, offering a complementary alternative to microscopy.

Similar to previous studies [54–56], our findings demonstrate that microscopic urinalysis (67.0% sensitivity and 73.2% specificity) and urine dipstick, particularly LE (59.8% sensitivity and 75.9% specificity), offer comparable diagnostic performance. These moderate performances suggest that both tests can complement each other to enhance diagnostic accuracy, with microscopy improving sensitivity, especially when combined with LE testing. While not as definitive as urine culture, these tools are useful for initial screening, particularly in resource-limited settings. However, their moderate sensitivity and specificity carry risks of missed diagnoses or unnecessary antibiotic use, highlighting the need for confirmatory testing. Given its superior sensitivity compared to the nitrite dipstick test, microscopy is more reliable for diagnosing UTIs when dipstick results are inconclusive. Despite its moderate accuracy (70.1%), microscopy's false-negative (33%) and false-positive (27.9%) rates reinforce the importance of confirmatory testing with urine culture.

While dipstick screening remains a useful point-of-care tool, its high false-negative rates highlight the need for a stepwise diagnostic approach that incorporates microscopy and urine culture to improve accuracy and prevent missed diagnoses [17, 57–62]. Current guidelines support this strategy, recommending culture-based confirmation in cases of diagnostic uncertainty or strong clinical suspicion, ultimately ensuring timely and appropriate management of UTIs [63, 64].

This study offers several strengths: It is the first of its kind nationally, utilizes a large sample size, and employs a comprehensive comparative approach while highlighting sex-based diagnostic differences that enhance its clinical relevance. Conducted in a real-world hospital setting, the findings deliver practical guidance for pediatric UTI diagnosis, particularly in resource-limited settings. This study has certain limitations, including its retrospective design, single-center scope, and potential selection bias, which may affect generalizability. Microscopy results could have been influenced by operator variability, and important confounding factors—such as stratification into hospital-acquired versus community-acquired infections, retroviral infection status, and other clinical variables—could not be explored. The absence of longitudinal follow-up limited our ability to assess long-term consequences such as misdiagnosis or delayed treatment. Inherent challenges of retrospective data, including possible errors in specimen handling and reporting, may also have contributed to inaccuracies. Finally, while only symptomatic or clinically suspected pediatric cases were included, classifying all culture-positive results as UTI means that the inclusion of asymptomatic bacteriuria cannot be entirely ruled out. This study recorded LE results only as positive or negative, precluding analysis of finer gradations and their correlation with WBC counts. While sufficient for assessing diagnostic accuracy, future studies incorporating LE levels could provide more detailed insights into test performance.

## 5. Conclusion

The combination of dipstick markers (nitrites and LE) offers a rapid and cost-effective approach for ruling in UTIs, particularly beneficial for initiating treatment while awaiting culture results in children. However, their limited ability to exclude UTIs, especially in symptomatic patients, and the reduced specificity when both markers are used together necessitate confirmatory diagnostics like urine culture or WBC microscopy. To improve diagnostic precision and reduce invasive procedures, future research should focus on discovering new urinary biomarkers, such as inflammatory proteins and molecular markers. Moreover, exploring diagnostic strategies that consider age and sex differences could further refine clinical practice.

## Figures and Tables

**Table 1:** Sex-based distribution of urine dipstick, WBC microscopy, and culture results among children ≤ 15 years at Tikur Anbessa Specialized Hospital, Addis Ababa (*N* = 224).

		**Males (%)**	**Females (%)**	**Total (%)**
Dipstick nitrites	Positive	16 (10.9)	8 (10.9)	24 (10.9)
	Negative	134 (89.1)	66 (89.1)	200 (89.1)
Dipstick leucocytes	Positive	84 (56)	32 (43.2)	116 (51.8)
	Negative	66 (44)	42 (56.8)	108 (48.2)
WBC microscope	Positive	79 (52.7)	26 (35.1)	105 (47.1)
	Negative	71 (47.3)	48 (64.9)	119 (52.9)
Culture	Positive	71 (47.3)	41 (55.4)	112 (50)
	Negative	79 (52.7)	33 (44.6)	112 (50)
Total		150 (66.7)	74 (33.3)	224 (100)

Abbreviations: TASH, Tikur Anbessa Specialized Hospital; WBC, white blood cell.

**Table 2:** Performance of urine dipstick leukocyte esterase and nitrite in comparison to culture results.

**Test**	**Result**	**Culture positive (%)**	**Culture negative (%)**	**Total (%)**	**Sensitivity (%)**	**Specificity (%)**	**PPV (%)**	**NPV (%)**	**Accuracy (%)**
Nitrite only	Positive	18 (16.1)	6 (5.4)	24 (10.7)	16.1	94.6	75.0	53.0	55.4
	Negative	94 (83.9)	106 (94.6)	200 (89.3)					
	Total	112 (100)	112 (100)	224 (100)					

Leukocyte only	Positive	67 (60.1)	27 (24.1)	94 (42.0)	59.8	75.9	71.3	65.4	67.9
	Negative	45 (40.2)	85 (75.9)	130 (58.0)					
	Total	112 (100)	112 (100)	224 (100)					

Either leukocyte or nitrite	Positive	69 (61.6)	29 (25.9)	98 (43.6)	61.6	74.1	70.4	65.4	67.9
	Negative	43 (38.4)	83 (74.1)	126 (56.3)					
	Total	112 (100)	112 (100)	224 (100)					

Leukocyte and nitrite	Positive	16 (14.3)	6 (5.4)	22 (9.8)	14.3	94.6	72.7	52.5	54.5
	Negative	96 (85.7)	106 (94.6)	202 (90.2)					

	Total	112 (100)	112 (100)	224 (100)					

Abbreviations: NPV, negative predictive value; PPV, positive predictive value.

**Table 3:** Urine WBC microscopy findings in relation to urine culture results among children under 15 years at TASH, Addis Ababa.

**Test**	**Result**	**Culture positive (%)**	**Culture negative (%)**	**Total (%)**	**Sensitivity (%)**	**Specificity (%)**	**PPV (%)**	**NPV (%)**	**Accuracy (%)**
WBC on microscopy	Positive	75 (67.0)	30 (27.9)	105 (46.9)	67.0	73.2	71.4	68.9	70.1
	Negative	37 (33.0)	82 (73.2)	119 (53.1)					
	Total	112 (100)	112 (100)	224 (100)					

Abbreviations: NPV, negative predictive value; PPV, positive predictive value; WBC, white blood cell.

**Table 4:** Performance of leukocyte esterase (dipstick) for detecting WBCs on microscopy.

**WBC on microscopy**	**Leukocyte esterase (dipstick) positive**	**Leukocyte esterase (dipstick) negative**	**Total**
Positive (WBCs present)	85 (80.8%)	27 (19.2%)	112 (100%)
Negative (no WBCs)	9 (7.5%)	103 (92.5%)	112 (100%)
Total	94 (42.0%)	130 (58.0%)	224 (100%)

Abbreviation: WBCs, white blood cells.

**Table 5:** Pearson correlation between leukocyte esterase (dipstick) and WBC count per high-power field (hpf) in microscopic examination.

**Variable**	**Dipstick leukocyte**	**WBC microscope**
Dipstick leukocyte	Pearson correlation	1
	Sig. (two-tailed)	—
	*N*	224

WBC microscope	Pearson correlation	0.741
	Sig. (two-tailed)	0.000
	*N*	224

## Data Availability

All relevant data underpinning the study's conclusions are included in the article. Additional data will be available from the corresponding authors upon reasonable request.

## Nomenclature

BSC: Bachelor of Science (nurses)
cfu/mL: colony-forming units per milliliter
CI: confidence interval
*E. coli*: *Escherichia coli*
hpf: high-power field
LE: leukocyte esterase
NPV: negative predictive value
PPV: positive predictive value
SPSS: Statistical Package for the Social Sciences
TASH: Tikur Anbessa Specialized Hospital
UTI: urinary tract infection
WBC: white blood cell
